# Noninvasively Assessed Portal Hypertension Grade Predicts Post-Hepatectomy Liver Failure in Patients With HepatocellCarcinoma: A Multicenter Study

**DOI:** 10.3389/fonc.2022.934870

**Published:** 2022-07-14

**Authors:** Jitao Wang, Zhanguo Zhang, Dong Shang, Jinlong Li, Chengyu Liu, Peng Yu, Mingguang Wang, Dengxiang Liu, Hongrui Miao, Shuang Li, Biao Zhang, Anliang Huang, Yewei Zhang, Shubo Chen, Xiaolong Qi

**Affiliations:** ^1^ Center of Portal Hypertension, Department of Radiology, Zhongda Hospital, School of Medicine, Southeast University, Nanjing, China; ^2^ Xingtai Key Laboratory of Precision Medicine for Liver Cirrhosis and Portal Hypertension, Xingtai People’s Hospital of Hebei Medical University, Xingtai, China; ^3^ Department of Hepatobiliary Surgery, Tongji Hospital Affiliated to Huazhong University of Science and Technology, Wuhan, China; ^4^ Department of General Surgery, The First Affiliated Hospital of Dalian Medical University, Dalian, China; ^5^ Department of Hepatobiliary Surgery, Fifth Medical Center of People’s Liberation Army of China (PLA) General Hospital, Beijing, China; ^6^ Department of Hepatobiliary Surgery, The Second Affiliated Hospital of Nanjing Medical University, Nanjing, China

**Keywords:** non-invasive diagnosis, liver resection, complication, portal hypertension, post-hepatectomy liver failure

## Abstract

**Purpose:**

To determine the predictive value of portal hypertension (PH) for the development of post-hepatectomy liver failure (PHLF) in patients with hepatocellular carcinoma (HCC).

**Patients and methods:**

This study enrolled a total of 659 patients with HCC that received hepatectomy as a first-line therapy. PH was classified as grade 0, 1, and 2 according to whether the indirect criteria for PH were met: 1) patients had obvious varicose veins and 2) splenomegaly was present and platelet count < 100 × 10^9^/L. The effects of each variable on the occurrence of PHLF were assessed using univariate and multivariate analyses.

**Results:**

PH grade 2 (odds ratio [OR] = 2.222, p = 0.011), higher age (OR = 1.031, p = 0.003), hepatitis C infection (OR = 3.711, p = 0.012), open surgery (OR = 2.336, p < 0.001), portal flow blockage (OR = 1.626, p = 0.023), major hepatectomy (OR = 2.919, p = 0.001), hyperbilirubinemia (≥ 17.2 μmol/L, OR = 2.113, p = 0.002), and high levels of alpha-fetoprotein (> 400n g/ml, OR = 1.799, p = 0.008) were significantly associated with PHLF occurrence. We performed a subgroup analysis of liver resection and found that the extent of liver resection and PH grade were good at distinguishing patients at high risk for PHLF, and we developed an easy-to-view roadmap.

**Conclusion:**

PH is significantly related to the occurrence of PHLF in patients who underwent hepatectomy. Noninvasively assessing PH grade can predict PHLF risk.

## Introduction

Hepatocellular carcinoma (HCC) is one of the common malignant tumors. Currently, the preferred treatment for HCC is radical liver resection. However, since the successful completion of the world’s first elective liver resection by German physician Langenbueh in 1888, the continual development of surgical techniques, liver resection instruments, and perioperative management has significantly improved the safety of hepatectomy, and the indications for hepatectomy have also expanded (1, 2). The perioperative mortality rate after liver resection has been reported to exceed 10% (3). Post-hepatectomy liver decompensation increases the likelihood of post-hepatectomy liver failure (PHLF), which has been shown to be the leading cause of death after hepatectomy (4). The morbidity rate and poor clinical outcomes of PHLF have created serious social and public health problems. Therefore, prediction of high-risk patients for PHLF remains a significant concern that needs to be addressed urgently.

Portal hypertension (PH) is one of the high-risk factors for the development of PHLF (5). Most HCC patients undergoing liver resection are often in the stage of compensated cirrhosis. The severity of PH can predict the occurrence of PHLF (6). Hepatic venous pressure gradient (HVPG) measurement is the gold standard for the diagnosis of PH, but the invasiveness, high technical difficulty, and radiation exposure involved hinder its clinical application. The current indirect indicators of PH (presence of esophagogastric varices, platelet count <100 × 10^9^/L, and spleen long diameter >12 cm) have been confirmed to predict the occurrence of PHLF (6–9). Previous studies were qualitative studies based on indirect criteria (i.e., presence or absence of PH) and could not further clarify the impact of preoperative PH severity on PHLF.

In previous guidelines for liver cancer, the presence of PH was specified as a contraindication for hepatectomy (10, 11). However, some studies have pointed out that even if PH is present before surgery, the poor prognosis after liver resection can be avoided when the extent of liver resection is reduced and treatment is timely (12, 13). Therefore, the relevance of PH as an independent factor in predicting complications such as PHLF and prognosis has been questioned (14). Several recent guidelines have modified the approach from considering PH as a contraindication for hepatectomy to a preoperative risk assessment that relies on a comprehensive evaluation system including PH, surgical approach, and extent of hepatectomy to select the best recipient (15, 16).

This study innovatively classified the severity of PH according to the preoperative clinical characteristic indicators and further clarified the predictive value of PH grading combined with the extent of hepatectomy in HCC patients with PHLF in a multicenter cohort of patients who underwent HCC resection.

## Methods

### Patient Selection

This study was a multicenter retrospective study, analyzing patients who underwent hepatectomy in Xingtai People’s Hospital, Fifth Medical Center of PLA General Hospital, First Affiliated Hospital of Dalian Medical University, and Huazhong University of Science and Technology Hospital from 2012 to 2020. The inclusion criteria were as follows (1): age ≥ 18 years; (2) patients with HCC diagnosed according to histopathology who underwent hepatectomy; and (3) Child-Pugh A-B grade, and Barcelona Clinic Liver Cancer (BCLC) A-B stage. The exclusion criteria were as follows: (1) patients with vascular invasion, extrahepatic metastases, or metastatic liver cancer; (2) patients with a history of radiofrequency/microwave ablation, interventional chemotherapy, radiotherapy, or targeted or immune-related anti-cancer therapy; (3) patients with severe heart, lung or renal insufficiency or other systemic malignant tumors; (4) patients who underwent combined surgery with other organs during the same period; and (5) patients with incomplete clinical data or were lost to follow-up. This study was a retrospective case-control study. All patients and their families were fully informed and provided written consent before surgery, which complied with medical ethics regulations. This study was conducted in accordance with the Declaration of Helsinki and was approved by the Institutional Review Board of Xingtai People’s Hospital (IRB ID 2022-006). The researchers only retrospectively analyzed de-identified data from patients.

### Surgical Technique

Hepatectomy and perioperative management are performed by experienced surgeons and nursing teams. In short, the surgeon choses to perform laparoscopy or laparotomy according to the location and size of the tumor, and completes the liver resection according to the standard procedures (17). Ultrasonic dissector or the clamp crushing technique were applied for parenchymal transection. At the second porta hepatis, the branches of the hepatic vein and the liver tissue were carried out by stapler hepatectomy. The Pringle method was used to block the blood flow into the liver when necessary. After checking for bleeding and bile leakage before closing, a biliary drain was placed.

### Data Collection

The baseline data of patients were collected, including clinical baseline data (age, sex, height, and weight), etiology of liver disease, whether complicated with liver cirrhosis, laboratory tests (liver function, blood routine, and coagulation), PH clinical features (maximum spleen diameter and degree of esophagogastric varices), tumor status (size/number and extent), surgical conditions (surgical method, extent of resection, intraoperative blood loss, whether intraoperative blood transfusion was used, etc.), and PHLF situation.

### Definition

Based on the recommendations made by the BCLC guidelines, PH was diagnosed in this study based on indirect criteria (6–9), specifically: [1] obvious varicose veins and [2] splenomegaly and platelet count (PLT) <100 × 10^9^/L. The PH grades were defined as grade 0: when neither feature is present; grade 1: when one feature is present; and grade 2: both features are present (18). The Child–Pugh score was based on criteria reported in the literature (19). The model for end-stage liver disease (MELD) score was calculated using the following formula (20): MELD = (0.957 × ln[creatinine, mg/dl] + 0.378 × ln[bilirubin, mg/dl] + 1.12 × ln[international normalized ratio] + 0.643) × 10. Major hepatectomy was defined as liver resection of at least three liver segments, and minor hepatectomy was defined as liver resection of less than three segments (16). Mortality was defined as death that occurred within 30 days of surgery.

### Diagnostic Criteria of PHLF

In this study, the International Study Group of Liver Surgery (ISGLS) criteria (21) were used to diagnose PHLF on the premise of excluding biliary obstruction, the total bilirubin (TBil) level, and international normalized ratio (INR). PHLF was diagnosed when the TBil level and INR were elevated compared to preoperative levels on or after postoperative day 5, according to the normal limits of the local laboratory. According to the ISGLS criteria (22), PHLF class A is defined as transient deterioration of liver function that does not require a change in clinical management, and PHLF class B is defined as deviation from routine postoperative clinical management without invasive treatment, PHLF grade C was defined as a patient requiring invasive treatment.

### Statistical Analysis

R (The R Foundation, https://www.r-project.org/) software was used for statistical analyses. Statistical descriptions of enumeration data were expressed as numbers (percentage), and rates were compared using a chi-squared test or Fisher’s exact test. Statistical descriptions of measurement data were expressed as medians (interquartile range) and compared using a rank-sum test. Univariate and multivariate logistic regression analyses were performed to determine whether each variable was an independent risk factor of PHLF. Items with a P-value < 0.1 in the univariate analysis were included in the multivariate analysis. To balance the baseline data, the three groups were compared after propensity score matching (PSM) analysis using the R software to remove selection bias. P < 0.05 was considered statistically significant.

## Results

### Baseline Data

**Figure 1:**
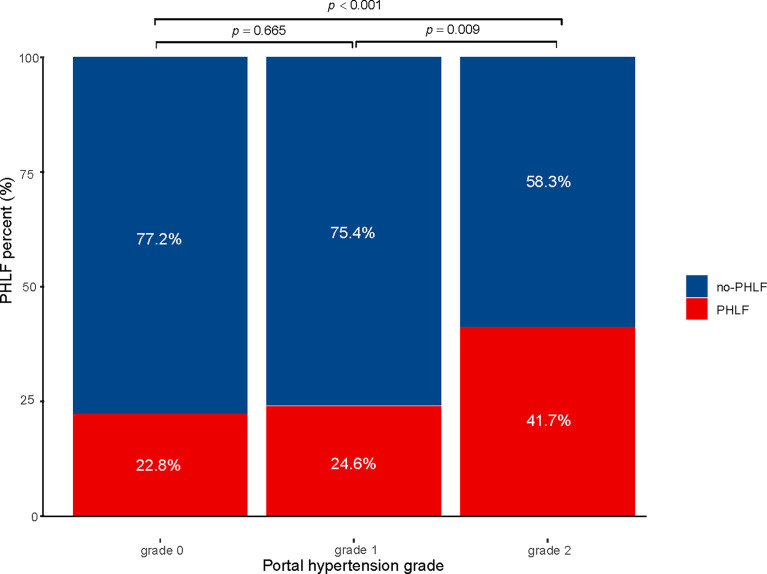
Incidence of PHLF with small-scale liver resection in patients with hepatocellular carcinoma PHLF, post hepatectomy liver failure.

Data pertaining to patients with histopathologically confirmed HCC who underwent hepatectomy were extracted and reviewed from a multicenter HCC database according to the inclusion criteria. In total, 42 cases were excluded because of extrahepatic distant metastasis (n = 10), preoperative anticancer treatment (n = 4), lost to follow-up (n = 23), and incomplete clinical data (n = 5). The remaining 659 patients were included in the final analysis. Among them, there were 254 cases in the Fifth Medical Center of the PLA General Hospital, 235 cases in the Affiliated Hospital of Huazhong University of Science and Technology, 115 cases in the First Affiliated Hospital of Dalian Medical University, and 55 cases in the Xingtai People’s Hospital. The median age was 53 (46–60) years and 559 patients were male (84.8%). The overall incidence of PHLF was 27.5% (181/659), of which PHLF grade A was 21.5% (142/659), PHLF grade B+C was 5.9% (39/659). The 30-day mortality in our cohort was 0.3% (2/659). The number of cases with PH grade 0, 1, and 2 were 390, 193, and 76, respectively. The incidence of PHLF in each group was 24.6%, 26.42%, and 44.7%, respectively (P < 0.001; **Figure 1**). Liver cirrhosis status, etiology of liver disease, surgical method of liver resection, extent of liver resection, Child–Pugh score, prothrombin time (PT), and PLT were statistically significant among the PH groups (P < 0.05). There were no differences in other baseline data between groups (**Table 1**).

**Table 1:** Comparisons of Characteristics between different portal hypertension grades.

Characteristics	Total (N= 659)	PH grade 0 (N= 390)	PH grade 1 (N= 193)	PH grade 2 (N= 76)	*P* value
Age, years	53 (46 - 60)	53 (46 - 60)	54 (47 - 60)	51 (43 - 58.2)	0.815
Gender					0.710
Female	100 (15.2)	60 (15.4)	31 (16.1)	9 (11.8)	
Male	559 (84.8)	330 (84.6)	162 (83.9)	67 (88.2)	
ASA classification					0.077
Grade 1	217 (32.9)	135 (34.6)	62 (32.1)	20 (26.4)	
Grade 2	404 (61.3)	239 (61.3)	118 (61.1)	47 (61.8)	
Grade 3	38 (5.8)	16 (4.1)	13 (6.8)	9 (11.8)	
Etiology					
HBV					0.060
NO	72 (10.9)	52 (13.3)	14 (7.3)	6 (7.9)	
YES	587 (89.1)	338 (86.7)	179 (92.7)	70 (92.1)	
HCV					0.134
NO	639 (97.0)	381 (97.7)	183 (94.8)	75 (98.7)	
YES	20 (3.0)	9 (2.3)	10 (5.2)	1 (1.3)	
Alcoholic					0.642
NO	654 (99.2)	387 (99.2)	192 (99.5)	75 (98.7)	
YES	5 (0.8)	3 (0.8)	1 (0.5)	1 (1.32)	
Others					**0.008**
NO	603 (91.5)	72 (94.7)	346 (88.7)	185 (95.8)	
YES	56 (8.5)	4 (5.3)	44 (11.3)	8 (4.2)	
HBV DNA					0.506
<500 Copys	339 (51.4)	202 (51.8)	94 (48.7)	43 (56.6)	
>500 Copys	320 (48.6)	188 (48.2)	99 (51.3)	33 (43.4)	
Cirrhosis status					**<0.001**
NO	81 (12.3)	67 (17.2)	12 (6.2)	2 (2.6)	
YES	578 (87.7)	323 (82.8)	181 (93.8)	74 (97.4)	
Surgical approach					**0.004**
Laparoscopy	279 (42.3)	179 (45.9)	63 (32.6)	37 (48.7)	
Open	380 (57.7)	211 (54.1)	130 (67.4)	39 (51.3)	
Extent of resection					**0.001**
Minor	589 (89.4)	334 (85.6)	183 (94.8)	72 (94.7)	
Major	70 (10.6)	56 (14.4)	10 (5.2)	4 (5.3)	
Hepatic inflow occlusion					0.508
NO	246 (37.3)	151 (38.7)	71 (36.8)	24 (31.6)	
YES	413 (62.7)	239 (61.3)	122 (63.2)	52 (68.4)	
Intraoperative blood loss					0.808
<400 ml	437 (66.3)	261 (66.9)	128 (66.3)	48 (63.2)	
≥400 ml	222 (33.7)	129 (33.1)	65 (33.7)	28 (36.8)	
Intraoperative transfution					0.078
NO	509 (77.2)	313 (80.3)	141 (73.1)	55 (72.4)	
YES	150 (22.8)	77 (19.7)	52 (26.9)	21 (27.6)	
Tumor number					0.710
Single	559 (84.8)	330 (84.6)	162 (83.9)	67 (88.2)	
Multipe(≥2)	100 (15.2)	60 (15.4)	31 (16.1)	9 (11.8)	
Tumor size, cm	4 (3 - 5)	4 (3 - 6)	3 (2 - 5)	3 (2 - 4)	0.477
ALT, U/L	31 (22 - 45)	30 (21 - 44)	32 (22 - 45)	33 (23 - 43)	0.420
AST, U/L	30 (23 - 43)	28 (22 - 40)	32 (25 - 47)	34 (26 - 46)	0.142
Albumin, g/L	40 (37 - 42.2)	40 (38 - 43)	39 (36 - 42)	38 (35 - 40)	0.092
Total bilirubin, μmol/L	14 (10 - 19)	13 (10 - 17)	14 (11 - 19)	17 (13 - 20)	0.314
Creatinine, μmol/L	75 (64 - 83)	75 (64 - 83)	75 (65 - 83)	77 (67 - 86)	0.635
PT, s	13 (12 - 14)	13 (11 - 14)	13 (12 - 14)	14 (13 - 15)	**0.013**
RBC, 10^12^/L	4.6 (4.2 - 4.9)	4.6 (4.3 - 4.9)	4.5 (4.2 - 4.9)	4.2 (3.8 - 4.7)	0.098
AFP, ng/ml	23 (7 - 384)	25.9 (6 - 447)	18 (8 - 162)	97 (12 - 611)	0.270
Platelet, 10^9^/L	140 (96 - 189)	165 (126 - 209)	116 (83 - 156)	59 (45 - 78)	**<0.001**
Child-Pugh class					**<0.001**
Grade A	622 (94.4)	376 (96.41)	184 (95.34)	62 (81.58)	
Grade B	37 (5.6)	14 (3.59)	9 (4.66)	14 (18.42)	
Meld score	5 (3 - 7)	4 (2 - 6)	5 (4 - 7)	7 (5 - 9)	0.183
PHLF					**0.002**
NO	478 (72.5)	294 (75.4)	142 (73.6)	42 (55.3)	
YES	181 (27.5)	96 (24.6)	51 (26.4)	34 (44.7)	

PH, portal hypertension; ASA, American Society of Anesthesiologists; HBV, Hepatitis B virus; HCV, Hepatitis C virus; ALT, alanine transaminase; AST, aspartate transaminase; PT, prothrombin time; RBC, red blood cell; AFP, a-fetoprotein level; MELD, Model For End-Stage Liver Disease; PHLF, post hepatectomy liver failure. Values with P < 0.05 in the tables are in bold.

### Univariate Analysis

Univariate analysis showed that PH grade 2, advanced age, positive hepatitis B viral DNA (> 500 copies), hepatitis C virus (HCV) infection, laparotomy, intraoperative blockade of portal blood flow, extensive hepatectomy, intraoperative bleeding (> 400 mL), multiple intrahepatic tumors, larger tumor diameter, alanine transaminase (ALT) level ≥ 40 U/L, aspartate transaminase (AST) level ≥ 40 U/L, albumin level < 35 g/L, TBil level ≥ 17.2 µmol/L, red blood cell count < 3.5×10^12^/L, alpha-fetoprotein (AFP) ≥ 400 ng/mL, Child–Pugh class B, and higher MELD scores were significantly associated with postoperative PHLF (**Table 2**).

**Table 2:** Factors Associated with Post Hepatectomy Liver Failure at Univariate and Multivariate Logistic Regression Analyses.

Characteristics	Univariable logistic regression		Multvariable logistic regression	
	0R (95%CI)	P value	0R (95%CI)	P value
Age, years	1.022 (1.004-1.04)	**0.017**	1.031 (1.010-1.052)	**0.003**
Gender, male	0.969 (0.609-1.578)	0.897		
ASA classification				
grade 2 vs grade1	0.619 (0.430-0.892)	**0.001**	0.805 (0.521-1.247)	0.329
grade 3 vs grade1	1.175 (0.562-2,381)	0.660	1.130 (0.484-2.557)	0.772
Etiology				
HBV, Yes	0.844 (0.501-1.464)	0.534		
HCV, Yes	3.372 (1.372-8.501)	**0.008**	3.711 (1.337-10.479)	**0.012**
Alcoholic, Yes	1.769 (0.232-10.76)	0.534		
others, Yes	1.062 (0.564-1.913)	0.846		
HBV DNA, >500 copy	1.540 (1.092-2.177)	**0.014**	1.384 (0.937-2.050)	0.103
Cirrhosis, Yes	1.623 (0.934-2.978)	0.099	1.093 (0.578-2.161)	0.790
Surgical approach, Open surgery	1.824 (1.277-2.627)	**0.001**	2.336 (1.470-3.789)	**<0.001**
Extent of resection, Major	2.175 (1.301-3.608)	**0.003**	2.919 (1.553-5.487)	**0.001**
Hepatic inflow occlusion, Yes	1.525 (1.062-2.209)	**0.024**	1.626 (1.075-2.488)	**0.023**
Intraoperative Blood loss, ≥400 ml	1.593 (1.117-2.268)	**0.010**	1.165 (0.773-1.747)	0.462
Intraoperative transfution, Yes	1.277 (0.853-1.892)	0.228		
Tumor number, Multipe	1.693 (1.074-2.638)	**0.021**	1.134 (0.678-1.871)	0.626
Tumor size, cm	1.082 (1.021-1.147)	**0.008**	1.052 (0.981-1.129)	0.154
ALT, ≥40 U/L	1.627 (1.137-2.325)	**0.008**	1.566 (0.968-2.530)	0.067
AST, ≥40 U/L	1.823 (1.268-2.614)	**0.001**	0.898 (0.542-1.474)	0.673
Albumin, <35 g/L	1.880 (1.152-3.035)	**0.010**	1.037 (0.565-1.871)	0.904
Total bilirubin, ≥17.2 μmol/L	2.499 (1.744-3.583)	**<0.001**	2.113 (1.308-3.423)	**0.002**
PT, ≥15s	1.674 (0.966-2.847)	0.061	1.514 (0.705-3.192)	0.280
RBC, <3.5*10^12^/L	3.766 (1.753-8.301)	**0.001**	1.959 (0.800-4.845)	0.140
AFP, ≥400 ng/ml	1.495 (1.016-2.186)	**0.039**	1.799 (1.162-2.780)	**0.008**
Child-Pugh score, (B class vs A class)	3.369 (1.723-6.660)	**<0.001**	2.233 (0.970-5.165)	0.058
MELD score	1.089 (1.025-1.158)	**0.006**	0.987 (0.910-1.071)	0.759
PH grade				
grade 1 vs grade0	1.100 (0.738-1.626)	0.636	1.010 (0.644-1.574)	0.963
grade 2 vs grade0	2.479 (1.487-4.116)	**<0.001**	2.222 (1.199-4.102)	0.011

PHLF, post hepatectomy liver failure; ASA, American Society of Anesthesiologists; HBV, Hepatitis B virus; HCV, Hepatitis C virus; ALT, alanine transamise; AST, aspartate transamise; PT, prothrombin time; RBC, red blood cell; AFP, a-fetoprotein level; MELD, Model For End-Stage Liver Disease; PH, portal hypertension. Values with P < 0.05 in the tables are in bold.

### Multivariate Analysis

Multivariate regression analysis revealed that PH grade 2 [odds ratio [OR] 2.222; 95% confidence interval [CI], 1.199-4.102], age (OR, 1.031; 95% CI, 1.012-1.052), HCV infection (OR, 3.711; 95% CI, 1.337-10.479), laparotomy (OR, 2.336; 95% CI, 1.470-3.789), hepatic blood loss (OR, 1.626; 95% CI, 1.075-2.488), extensive hepatectomy (OR, 2.919; 95% CI, 1.553-5.487), TBil level ≥ 17.2 µmol/L (OR, 2.113; 95% CI, 1.308-3.423), and AFP level > 400 ng/mL (OR, 1.799; 95% CI, 1.162-2.780) were independent risk factors for postoperative PHLF (**Table 2**).

### Propensity Score Matching (PSM)

To balance differences in baseline variables among the three PH grade groups, we performed propensity score matching (PSM). After PSM, there were no statistically significant differences in baseline variables other than PLT (one of the defining criteria for PH grades) among the three subgroups (**Table 3**). Further univariate and multivariate analyses reconfirmed that PH grade 2 was an independent risk factor for PHLF (**Table 4**).

**Table 3:** Comparisons of Characteristics between different portal hypertension grades after Propensity Score Matching.

Characteristics	Total (N= 186)	PH grade 0 (N= 62)	PH grade 1 (N= 62)	PH grade 2 (N= 62)	*P value*
Age, years	52 (45 - 58)	51 (45 - 56)	54 (45 - 57.8)	53.5 (46 - 58.8)	0.178
Gender					0.882
Female	22 (11.83)	6 (9.68)	8 (12.9)	8 (12.9)	
Male	164 (88.17)	56 (90.32)	54 (87.1)	54 (87.1)	
ASA classification					0.711
Grade 1	47 (25.27)	14 (22.58)	15 (24.19)	18 (29.03)	
Grade 2	121 (65.05)	44 (70.97)	40 (64.52)	37 (59.68)	
Grade 3	18 (9.68)	4 (6.45)	7 (11.29)	7 (11.29)	
Etiology					
HBV					0.732
NO	17 (9.14)	7 (11.29)	4 (6.45)	6 (9.68)	
YES	169 (90.86)	55 (88.71)	58 (93.55)	56 (90.32)	
HCV					0.999
NO	182 (97.85)	60 (96.77)	61 (98.39)	61 (98.39)	
YES	4 (2.15)	2 (3.23)	1 (1.61)	1 (1.61)	
Alcoholic					0.999
NO	183 (98.39)	61 (98.39)	61 (98.39)	61 (98.39)	
YES	3 (1.61)	1 (1.61)	1 (1.61)	1 (1.61)	
Others					0.776
NO	176 (94.62)	58 (93.55)	60 (96.77)	58 (93.55)	
YES	10 (5.38)	4 (6.45)	2 (3.23)	4 (6.45)	
HBV DNA					0.900
<500 Copys	95 (51.08)	32 (51.61)	30 (48.39)	33 (53.23)	
>500 Copys	91 (48.92)	30 (48.39)	32 (51.61)	29 (46.77)	
Cirrhosis status					0.999
NO	4 (2.15)	1 (1.61)	1 (1.61)	2 (3.23)	
YES	182 (97.85)	61 (98.39)	61 (98.39)	60 (96.77)	
Surgical approach					0.688
Laparoscopy	83 (44.62)	30 (48.39)	25 (40.32)	28 (45.16)	
Open	103 (55.38)	32 (51.61)	37 (59.68)	34 (54.84)	
Extent of resection					0.999
Minor	175 (94.09)	58 (93.55)	59 (95.16)	58 (93.55)	
Major	11 (5.91)	4 (6.45)	3 (4.84)	4 (6.45)	
Hepatic inflow occlusion					0.891
NO	65 (34.95)	20 (32.26)	23 (37.1)	22 (35.48)	
YES	121 (65.05)	42 (67.74)	39 (62.9)	40 (64.52)	
Intraoperative blood loss					0.999
<400 ml	118 (63.44)	39 (62.9)	39 (62.9)	40 (64.52)	
≥400 ml	68 (36.56)	23 (37.1)	23 (37.1)	22 (35.48)	
Intraoperative transfution					0.743
NO	138 (74.19)	48 (77.42)	44 (70.97)	46 (74.19)	
YES	48 (25.81)	14 (22.58)	18 (29.03)	16 (25.81)	
Tumor number					0.962
Single	162 (87.1)	53 (85.48)	55 (88.71)	54 (87.1)	
Multipe(≥2)	24 (12.9)	9 (14.52)	7 (11.29)	8 (12.9)	
Tumor size, cm	3 (2 - 4)	3.5 (2.7 - 4.9)	3 (2 - 3.6)	3 (2 - 4)	0.515
ALT, U/L	34 (23 - 45)	34 (25 - 51.8)	36 (23 - 50.5)	33 (23 - 43)	0.425
AST, U/L	32 (24 - 47)	31.5 (23 - 46.8)	35.5 (25 - 52.8)	32.5 (24.2 - 44)	0.828
Albumin, g/L	38.8 (35 - 41)	39.2 (37.1 - 42.1)	37.8 (34.1 - 40.9)	38 (35.5 - 40.2)	0.379
Total bilirubin, μmol/L	16.8 (11.9 - 21.3)	16.4 (11.9 - 21)	17.9 (12.1 - 25.1)	16.6 (11.8 - 19.8)	0.566
Creatinine, μmol/L	77 (65 - 84)	77.5 (65 - 84)	76.5 (62.5 - 83)	77.5 (69.2 - 86.5)	0.614
PT, s	13.4 (12.3 - 14.8)	13.6 (12 - 14.8)	13.1 (12.1 - 14.2)	13.5 (12.5 - 14.8)	0.854
RBC, 10^12^/L	4.4 (4 - 4.8)	4.5 (3.9 - 4.9)	4.3 (4.1 - 4.7)	4.4 (3.9 - 4.8)	0.479
AFP, ng/ml	60.7 (12.1 - 619.7)	159.1 (16 - 773.2)	37.8 (7.8 - 527.4)	37.1 (12.1 - 540.1)	0.449
Platelet, 10^9^/L	90.5 (65.6 - 149)	160.5 (114.5 - 176)	92 (71.8 - 149)	63.5 (50.1 - 81)	**0.006**
Child-Pugh class					0.652
Grade A	165 (88.71)	54 (87.1)	57 (91.94)	54 (87.1)	
Grade B	21 (11.29)	8 (12.9)	5 (8.06)	8 (12.9)	
Meld score	6.2 (4.5 - 8.2)	6.2 (4.7 - 8.2)	6.2 (4.8 - 8.3)	6.4 (4.3 - 8.1)	0.587
PHLF					**0.041**
NO	131 (70.43)	48 (77.42)	47 (75.81)	36 (58.06)	
YES	55 (29.57)	14 (22.58)	15 (24.19)	26 (41.94)	

PH, portal hypertension; ASA, American Society of Anesthesiologists; HBV, Hepatitis B virus; HCV, Hepatitis C virus; ALT, alanine transaminase; AST, aspartate transaminase; PT, prothrombin time; RBC, red blood cell; AFP, a-fetoprotein level; MELD, Model For End-Stage Liver Disease; PHLF, post hepatectomy liver failure. Values with P < 0.05 in the tables are in bold.

**Table 4:** Factors Associated with Post Hepatectomy Liver Failure at Univariate and Multivariate Logistic Regression Analyses after Propensity Score Matching.

Characteristics	Univariable logistic regression		Multvariable logistic regression	
	0R (95%CI)	*P* value	0R (95%CI)	*P* value
Age, years	1.059 (1.022-1.101)	**0.002**	1.05 (1.008-1.097)	**0.022**
Gender, male	1.491 (0.555-4.736)	0.456		
ASA classification	1.108 (0.532-0.707)	0.268		
grade 2 vs grade1	1.107 (0.532-2.400)	0.789		
grade 3 vs grade1	1.308 (0.388-4.153)	0.653		
Etiology				
HBV, Yes	0.567 (0.206-1.641)	0.276		
HCV, Yes	2.434 (0.286-20.717)	0.38		
Alcoholic, Yes	1.194 (0.055-12.722)	0.886		
others, Yes	1.634 (0.404-5.961)	0.461		
HBV DNA, >500 copy	1.698 (0.902-3.236)	0.103		
Cirrhosis, Yes	6778148.135 (0-)	0.99		
Surgical approach, Open surgery	1.058 (0.562-2.008)	0.861		
Extent of resection, Major	4.63 (1.337-18.343)	**0.018**	4.154 (1.032-18.616)	**0.048**
Hepatic inflow occlusion, Yes	1.293 (0.666-2.578)	0.455		
Intraoperative Blood loss, ≥400 ml	1.104 (0.571-2.107)	0.766		
Intraoperative transfution, Yes	1.114 (0.536-2.246)	0.767		
Tumor number, Multipe	1.857 (0.751-4.459)	0.169		
Tumor size, cm	1.039 (0.868-1.236)	0.664		
ALT, ≥40 U/L	1.829 (0.956-3.499)	0.067	1.643 (0.668-4.048)	0.277
AST, ≥40 U/L	1.968 (1.031-3.764)	**0.04**	1.058 (0.408-2.689)	0.907
Albumin, <35 g/L	2.831 (1.382-5.815)	**0.004**	1.806 (0.751-4.339)	0.184
Total bilirubin, ≥17.2 μmol/L	1.806 (0.959-3.446)	0.069	1.794 (0.846-3.874)	0.13
PT, ≥15s	1.761 (0.825-3.691)	0.137		
RBC, <3.5*10^12^/L	2.617 (0.914-7.507)	0.069	1.259 (0.33-4.51)	0.727
AFP, ≥400 ng/ml	0.693 (0.339-1.366)	0.3		
Child-Pugh score, (B class vs A class)	3.783 (1.499-9.876)	**0.005**	1.661 (0.543-5.149)	0.371
MELD score	1.053 (0.943-1.178)	0.359		
PH grade	1.094 (0.475-0.514)	0.212		
grade 1 vs grade0	1.094 (0.475-2.535)	0.832	0.928 (0.37-2.331)	0.873
grade 2 vs grade0	2.476 (1.149-5.513)	**0.023**	2.559 (1.106-6.151)	**0.031**

PHLF, post hepatectomy liver failure; ASA, American Society of Anesthesiologists; HBV, Hepatitis B virus; HCV, Hepatitis C virus; ALT, alanine transamise; AST, aspartate transamise; PT, prothrombin time; RBC, red blood cell; AFP, a-fetoprotein level; MELD, Model For End-Stage Liver Disease; PH, portal hypertension. Values with P < 0.05 in the tables are in bold.

### Subgroup Analysis

**Figure 2:**
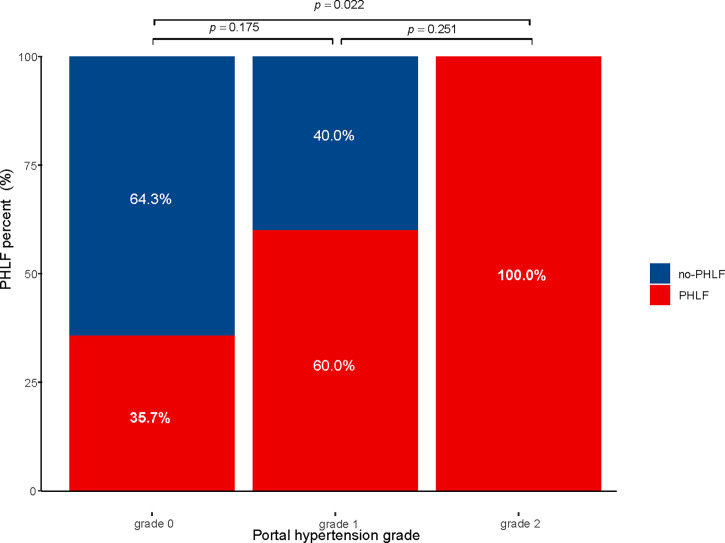
Incidence of PHLF with extensive liver resection in patients with hepatocellular carcinoma PHLF, post hepatectomy liver failure.

**Figure 3:**
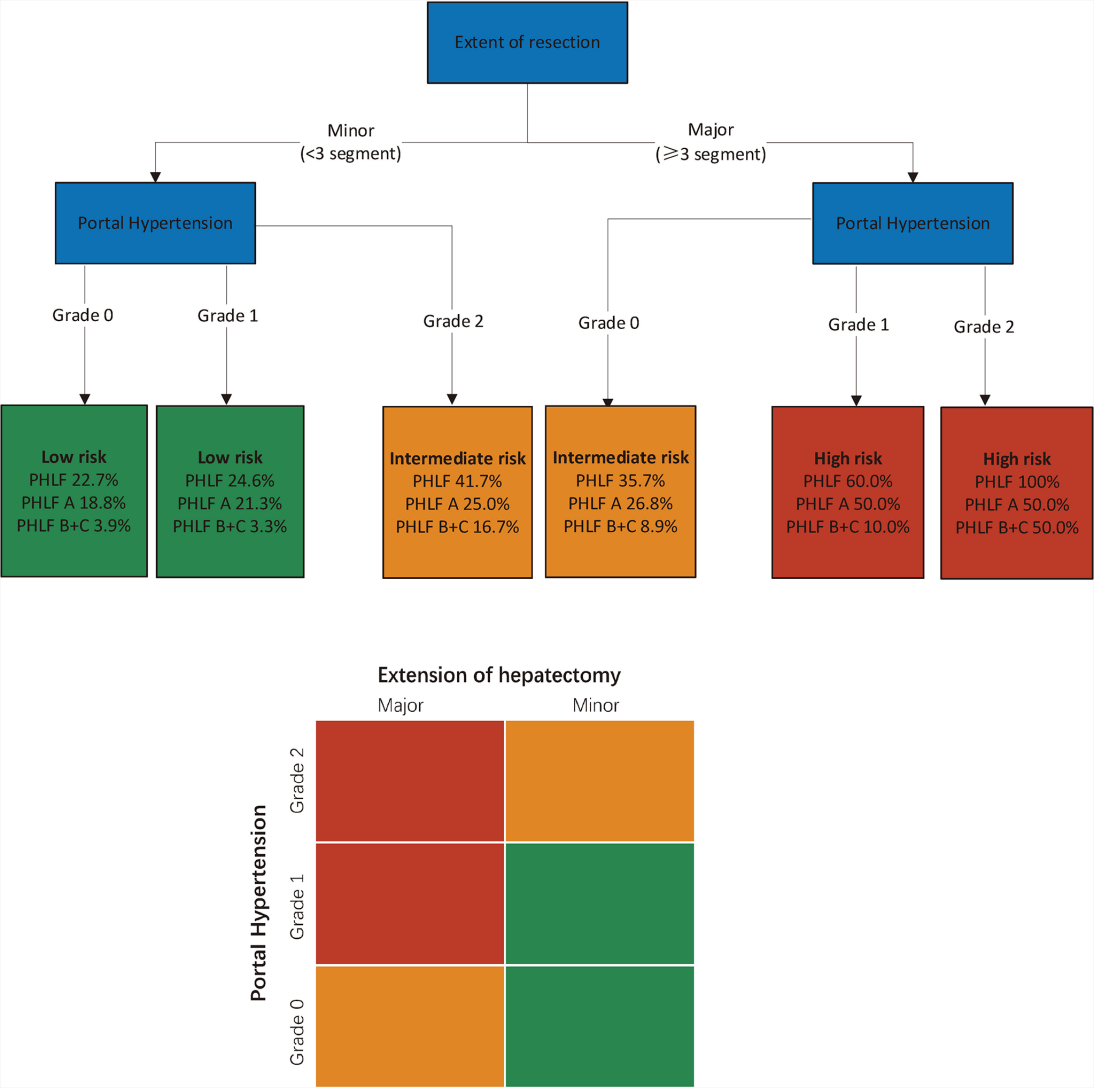
Multi-parametric assessment of the risk of PHLF in patients with hepatocellular carcinoma. Simplified decisional algorithm identifying high (red), intermediate (yellow) and low (green) risk of PHLF, according to a hierarchic interaction of the two independent risk factors: portal hypertension, and extent of resection. Major hepatectomy, hepatectomy range at least 3 liver segments; minor hepatectomy, hepatectomy range less than 3 segments. PHLF, post hepatectomy liver failure.

Subgroup analysis was performed according to the extent of liver resection. In the small-scale liver resection cohort, the incidence of postoperative PHLF in patients with PH grade 0, 1, and 2 was 24.6%, 26.4, and 44.7%, respectively (**Figure 1**). In patients in the extensive liver resection cohort, the trend was more obvious, and the incidence of PHLF in patients with PH grade 0, 1, and 2 was 35.7%, 60%, and 100%, respectively (**Figure 2**). We stratified patients at risk of developing PHLF according to the extent of liver resection and grade of PH and developed a surgeon-friendly roadmap for PHLF prediction (**Figure 3**).

## Discussion

Our study showed that the severity of PH as assessed by noninvasive PH grade assessed by preoperative noninvasive methods is a useful predictive marker of PHLF in patients with HCC who underwent liver resection. High-risk PHLF patients can be well identified according to the extent of liver resection and PH grade.

PHLF refers to a group of clinical syndromes characterized by abnormal liver function, ascites, jaundice, and hepatic encephalopathy after hepatectomy. Its primary mechanism involves liver resection that leads to serious dysfunction of liver synthesis, decomposition, enzyme metabolism, and biotransformation (23, 24). As one of the serious complications after hepatectomy, PHLF has been proven to be the leading cause of death after hepatectomy (4, 24). In a previous large clinical experiment, > 70% of all deaths in patients with indications for liver resection met the criteria for PHLF, and the fatality rate of patients with PHLF was >50% (25). Therefore, prediction of high-risk patients for PHLF remains an urgent clinical issue.

There is no unified and standardized definition for the diagnosis of PHLF. Currently, the main diagnostic criteria include the “50-50” and ISGLS criteria. The “50-50 criteria,” which predicts PHLF based on changes in PT and TBil, was first proposed by Balzan in 2005 after a statistical analysis of the clinical data of 775 patients who underwent liver resection (26). This method allows for easy calculation; the diagnostic specificity reaches 97.7%, but its sensitivity is only 69.6%, limiting its wide application. ISGLS redefined PHLF in 2011 and predicts PHLF based on whether the liver’s ability to maintain synthesis, excretion, and detoxification deteriorates after hepatectomy. The specific diagnostic criteria are to detect the levels of TBil and INR on the 5th postoperative day or later. PHLF can be diagnosed when TBil and INR levels are elevated compared to preoperative levels (21). The PHLF definition of ISGLS allows for calculation and comparison, making it widely used and gradually becoming the standardized definition of PHLF (24, 27, 28). Therefore, this study used the ISGLS-PHLF standard.

Previous studies have shown that the severity of PH is an independent predictor of decompensation and increased mortality (29). The EASL guidelines point out that, different from non-cirrhotic patients who are the first choice for surgical treatment, preoperative PH in patients with liver cirrhosis is an important influencing factor for the choice of surgical resection (16). With improvements in operator ability, more refined management of PH evaluation of patients during the perioperative period, and consideration of factors such as resection volume and liver function, the presence of PH is no longer a contraindication for hepatectomy. However, PH still seriously affects the prognosis and is inextricably linked to the occurrence of PHLF (6). Considering that HVPG has not yet been widely adopted in clinical practice, PH is still currently considered as a surrogate standard. Previous studies have qualitatively determined whether the presence of PH predicts postoperative complications in patients with HCC (6, 30, 31). A single-center study of 190 patients with Child-Pugh A HCC demonstrated that PH severity independently predicted the risk of PHLF(OR 3 24, 95% CI 1.38 to 7.65; P = 0.007) (32). However, because this study was developed based on a single center, so its generalizability needs to be verified in multiple centers. In addition, in the study, only 190 patients with HCC were enrolled, and all patients underwent laparotomy. Our multicenter study enrolled more HCC patients from different regions in China, and 42.3% (279/659) of the patients underwent laparoscopic surgery.

This study divided PH into three grades according to the clinical characteristics. Our study found that as the grade of PH increased, HCC patients were more likely to have liver cirrhosis, lower platelet levels, and worse liver function and coagulation function, and were more likely to undergo small-scale liver resection. This is consistent with the clinical characteristics of this population. The results showed that regardless of resection size, the PH grade positively correlated with the incidence of postoperative PHLF (p = 0.002). The same finding was also made in the subgroup analysis based on the extent of liver resection. Multivariate regression analysis showed that PH grade was an independent risk factor for PHLF.

There is a significant positive correlation between PH grade and PH after hepatectomy, and these phenomena may be caused by many complex and interacting factors. In terms of pathology, patients with liver cirrhosis have different mechanisms leading to inflammation and necrosis of liver tissue and the formation of fibrotic nodules, resulting in distorted and closed hepatic vascular structure. This histological state results in increased resistance to portal blood flow, which can lead to PH (33). We noted that the incidence of postoperative PHLF reached 100% in patients with PH grade 2 and 60% in patients with PH grade 1 who underwent extensive liver resection. Under similar portal pressure levels, extensive resection is more traumatic and requires higher liver function reserve; the risk of postoperative PHLF is thus greatly increased. This suggests that for patients with severe PH, adequate liver function and systemic status evaluation should be performed before undertaking extensive liver resection and consideration should be given to improving the patient’s general condition to improve the prognosis before surgery or other treatment. This conclusion is consistent with the EASL guidelines for surgical treatment of liver cancer with cirrhosis (16). On the other hand, similar conclusions could be drawn from the small-scale liver resection subgroup. Surgical resection is not an absolute contraindication even if with severe PH. Under the premise of adequate preoperative evaluation and preparation, small-scale liver resection is feasible, but it requires more careful perioperative management to effectively maintain the patient prognosis. Hepatectomy remains the relatively preferred treatment modality for those patients.

The use of liver resection extent and PH grade to assess the risk of PHLF is a practical method that can facilitate individual treatment decisions for patients with HCC based on the potential risk of PHLF. Moreover, the evaluation of PH grades for patients with HCC undergoing elective liver resection only requires common parameters, such as routine blood tests and preoperative computed tomography or gastroscopy, which is more practical for primary medical institutions. A roadmap for assessing PHLF based on PH grade and extent of liver resection is a useful tool for optimizing patient selection for liver resection and improving current treatment strategies.

Our study has some limitations. First, this is a multicenter retrospective study in China with a high proportion of patients with HBV infection. More patients with HCC with other etiologies of liver disease need to be prospectively included in the future to confirm our conclusions. Second, PH is indirectly diagnosed by clinical criteria and not by HVPG. Whether PH ratings accurately reflect HVPG values has not been fully validated. Finally, first, considering the retrospective nature of our study, part of data was incompletely recorded. Owing to the lack of other perioperative complications other than PHLF and long-term follow-up data, the relationship between PH grade and other perioperative complications and overall survival of patients with HCC who underwent hepatectomy requires additional studies. A large-scale, prospective multicenter study with external verification cohorts remains needed.

## Conclusion

PH is an independent risk factor for predicting the occurrence of PHLF, and the noninvasive methods of assessing PH grade may be a useful predictor of PHLF in patients with HCC after hepatectomy.

## Data Availability Statement

The raw data supporting the conclusions of this article will be made available by the authors, without undue reservation.

## Ethics Statement

The studies involving human participants were reviewed and approved by Institutional Review Board of Xingtai People’s Hospital (IRB ID 2022-006). The patients/participants provided their written informed consent to participate in this study.

## Author Contributions

JW and XQ contributed to conception and design of the study. ZZ, DS, JL, BZ, AH, and PY carried out the data curation. JL and CL performed the statistical analysis. MW, DL, HM and SL carried out the methodology. JW and YZ wrote the manuscript. YZ and SC carried out the project administration. All authors contributed to manuscript revision, read, and approved the submitted version.

## Conflict of Interest

The authors declare that the research was conducted in the absence of any commercial or financial relationships that could be construed as a potential conflict of interest.

## Publisher’s Note

All claims expressed in this article are solely those of the authors and do not necessarily represent those of their affiliated organizations, or those of the publisher, the editors and the reviewers. Any product that may be evaluated in this article, or claim that may be made by its manufacturer, is not guaranteed or endorsed by the publisher.
